# Adaptive Collaboration Between the Emergency Department and Neonatal Intensive Care to Treat a 16-Month-Old in Sepsis-Related Hemolytic Anemia with a Hemoglobin of 1.7 g/dL: A Case Report

**DOI:** 10.3390/pediatric18020048

**Published:** 2026-04-01

**Authors:** Matvei A. Mozhaev, Samuel J. Thomas, Evfrosiniia A. Mozhaeva, Vraj S. Patel, Mia N. Aboukhaled, Antonia Bartlett, Muhammad Ansari, Brooke N. Shook, Mark M. Walsh

**Affiliations:** 1 Department of Anesthesiology, Intensive Care, and Emergency Medical Services, Medical University Astana, Astana 010000, Kazakhstan; 2 Department of Emergency Medicine, Indiana University School of Medicine, South Bend, IN 46617, USA; 3 Department of Emergency Medicine, Saint Joseph Regional Medical Center, Mishawaka, IN 46545, USA; 4 Department of Internal Medicine/Pediatrics, Indiana University School of Medicine, Indianapolis, IN 46202, USA; 5 Department of Neonatal Intensive Care, Saint Joseph Regional Medical Center, Mishawaka, IN 46545, USA

**Keywords:** autoimmune hemolytic anemia, sepsis, blood transfusion, mechanical ventilation, case report

## Abstract

**Background/Objectives**: An 8-kg, 16-month-old child was brought to the emergency department of a regional community hospital with shallow respirations. Due to her pallor and the diluted appearance of the first blood sample, the emergency physician suspected sepsis associated with severe anemia. Her first laboratory results revealed a hemoglobin of 1.7 g/dL. Subsequent laboratory data revealed positive fibrin split products and hypofibrinogenemia with reticulocytosis. Because this regional community hospital did not have a pediatric intensivist, the emergency physician instead consulted a neonatal intensivist for guidance. **Methods**: A femoral intraosseous line was placed to allow aggressive massive transfusion. After consultation with the neonatal intensivist, packed red blood cells were transfused at a rate of 30 mL/kg/h. After transfusion, the patient became agitated and required repeated paralytic, sedative, and analgesic boluses of succinylcholine, ketamine, midazolam, dexmedetomidine, and fentanyl, with fentanyl and dexmedetomidine drips. The patient arrived at a tertiary care center 13 h after admission. **Results**: At the tertiary care center, the patient was weaned off the drips and was theorized to have secondary autoimmune hemolytic anemia due to sepsis after positive direct and indirect Coombs test. She was treated with a course of antibiotics, including cefepime and vancomycin, without steroids or immunotherapy. Five months later, her hemoglobin had returned to 12.1 g/dL, and she tested negative on direct and indirect Coombs test. **Conclusions**: This case highlights the importance of collaboration between and within departments to successfully manage pediatric hemostatic resuscitation.

## 1. Introduction

Anemia is a worldwide public health concern among children. Its causes are abundant, including iron deficiency, chronic or infectious diseases, and inherited blood disorders [1]. For children less than five years old, it has been classified by severity as mild (hemoglobin = 10.0–10.9 g/dL), moderate (hemoglobin = 7.0–9.9 g/dL), and severe (hemoglobin < 7.0 g/dL) [2]. Anemia during the first 2 years of life can lead to complications such as reduced cognitive development, lowered immunity, delayed growth, and recurrent infections [3].

A special, rare type of anemia is autoimmune hemolytic anemia (AIHA), which is an immune-hematological disorder with premature hemolysis caused by autoantibodies’ action against erythrocyte surface antigens. In children, it predominates in males, and it is a rare disease with an estimated annual incidence of 0.2 new cases per 100,000 inhabitants under 20 years of age [4]. Transient and intense hemolysis caused by AIHA can induce severe anemia, especially among children under 10 years of age [5].

Extreme cases of anemia necessitate urgent blood transfusion resuscitation, yet in pediatric settings, this intervention poses significant risks of complications. Massive transfusion (MT) and MT protocols (MTPs) are still poorly defined in pediatric and neonatal settings [6,7]. Complications arising from pediatric MT can include the transmission of a viral, bacterial, or other microbial infection, as well as transfusion-associated circulatory overload (TACO) [6,8].

For severe cases of pediatric anemia, the decision to administer MT is determined by adaptation, adoption, or adjustment of existing pediatric MTPs through a neonatal/developmental hemostasis lens [7]. In the context of the heterogeneous approaches for pediatric MT, our case stresses the benefits of interdepartmental collaboration between emergency and neonatal intensive care specialists for a non-neonate in the absence of pediatric intensive care specialists. Pediatric intensive care specialists still are not present in all communities because of limited workforce and resources [9]. This places children at a particular risk in emergency situations given differences in disease in comparison to adults [10]. The lack of a pediatric intensive care specialist in the community hospital in this case led the emergency physician to find a replacement in a neonatal intensive care specialist for a non-neonatal case. Sustained interdisciplinary collaboration between emergency medicine, neonatal intensive care, pharmacy, nursing, and respiratory therapy helped subdue potential silo boundaries typical between hospital departments.

## 2. Case Presentation

A 16-month-old was brought to an emergency department (ED) in the United States by her parents with chief complaints of whining, restlessness, and lack of appetite. She was born prematurely at 30 weeks with an Apgar score of 7 and, after spending six weeks in the neonatal intensive care unit, was discharged in good health. The only residual effect of prematurity was a weight of 8 kg, which placed the patient below the fifth percentile, while her twin brother was in the 50th percentile for weight. Otherwise, the child met all other developmental markers and was in good health until three weeks prior. The child had presented to the ED three separate times in the previous month with symptoms of upper respiratory tract infection. The child was well enough to go to daycare with her twin brother within five days leading up to the ED visit, but in the last two days, she could not keep any food down, had diarrhea, and became lethargic, leading her parents to bring her to the ED.

The physical exam revealed a temperature of 35.3 °C, heart rate of 152, respiratory rate of 30, blood pressure of 87/60, and peripheral oxygen saturation of 94%. The child appeared toxic but was crying, made eye contact, and withdrew from attempts to put on high-flow O_2_. The fontanel was closed. The conjunctivae were very pale, and pupils were small. The neck was supple, the chest was clear, and the abdomen was soft and nontender with no splenomegaly or hepatomegaly. The patient could move all four extremities, and the toes were downgoing. It was also noted during the physical exam that the patient was pale when compared to her twin brother.

The immediate impression was that this was a septic child. It was noted by the staff that when the blood was drawn, it looked diluted. An intravenous (IV) line was secured, and the patient was given a 20 mL/kg bolus of saline. It was noted on a point-of-care glucose test that the patient’s blood sugar was very low (<10 mg/dL), so the patient was given a bolus of 25% dextrose in water. The patient was immediately given vancomycin and ceftriaxone. The patient’s breathing became much shallower; therefore, it was decided that this patient needed immediate endotracheal intubation. The patient was given 12 mg ketamine and 12 mg succinylcholine intravenously, and the patient was successfully intubated on the first pass, using the video laryngoscope MAC 3 blade and a #4 cuffed ET tube with stylet. The patient was then connected to a Neonatal/Pediatric (Neo/Peds) ventilator on pressure control/assist control (PC/AC) with a positive end-expiratory pressure/continuous positive airway pressure of 5 cm H_2_O, set respiratory rate of 20, inspiratory rise time of 0.75 s, and fraction of inspired oxygen of 30%. The chest X-ray revealed that tube placement was appropriately placed just above the carina.

Laboratory results of the samples drawn before the saline bolus were delayed by a longstanding protocol of withholding severely abnormal results until confirmation with a repeat blood draw could be performed. Since the labs would not report on the computer, the laboratory results were only reported to the emergency physician after repeated, lengthy conversations with the laboratory personnel. The first labs that were reported to the emergency physician were a hemoglobin of 1.7 g/dL and a hematocrit of 4.7%. These results corroborated the pallor of the child when compared to her twin brother and the nurse’s note that the blood seemed diluted. Based on the extremely low hemoglobin and hematocrit, the emergency physician ordered an MT from the blood bank to be given at a rate of 30 mL/kg O negative packed red blood cells (PRBCs) over 1 h. This was given through an intraosseous (IO) line placed in the distal right femur with a power drill–driven IO access device with a 45-mm needle, proximal to the femoral diaphysis. The neonatal intensivist was consulted early during the care of this patient because of the emergency physician’s need to establish the safest dose of PRBCs to give to a septic child with an extremely low hemoglobin. Periodically, during the resuscitation, either through direct communication with the neonatal intensivist or through the respiratory therapists who were also working in the neonatal intensive care unit, the emergency physician was able to avail himself of their unique expertise, without the presence of a pediatric intensivist.

The initial complete blood count (CBC) supported the diagnosis of severe sepsis with a white blood cell (WBC) count of 59,700 per µL, platelet count of 463,000 per µL, and mean corpuscular volume (MCV) of 147 fL. The pathologist and emergency physician subsequently reviewed the blood smear, which revealed macrocytes with anisopoikilocytosis, polychromasia, nucleated RBCs, and adequate WBCs with myeloid left shift and toxic change. The smear also revealed occasional atypical and activated lymphoid cells, rare plasmacytoid cells, and smudge cells, which confirmed the diagnosis of hemolysis. Subsequently, the elevated MCV was a manifestation of the high number of smudge cells and nucleated red blood cells, which in turn were a response of the hemolysis by the bone marrow producing immature red cells into the peripheral blood.

Other laboratory data returned after transfusion had begun. A summary of relevant labs is presented in Table 1. Significant findings included a bilirubin of 1.4 mg/dL. Blood urea nitrogen and creatinine were normal, but procalcitonin was elevated at 0.71 ng/mL, as was C-reactive protein at 30.4 mg/L. The patient’s electrolytes were unremarkable, except for a bicarbonate of 6 mmol/L. Urinalysis was normal. Initial arterial blood gas after endotracheal intubation revealed metabolic acidosis with a pH of 7.09, CO_2_ partial pressure of 13 mmHg, and O_2_ partial pressure of 144 mmHg. The hypothermia with metabolic acidosis, as evidenced by the very low bicarbonate, and hypoglycemia supported the initial diagnosis of severe sepsis combined with severe hemolytic anemia.

**Figure 1:**
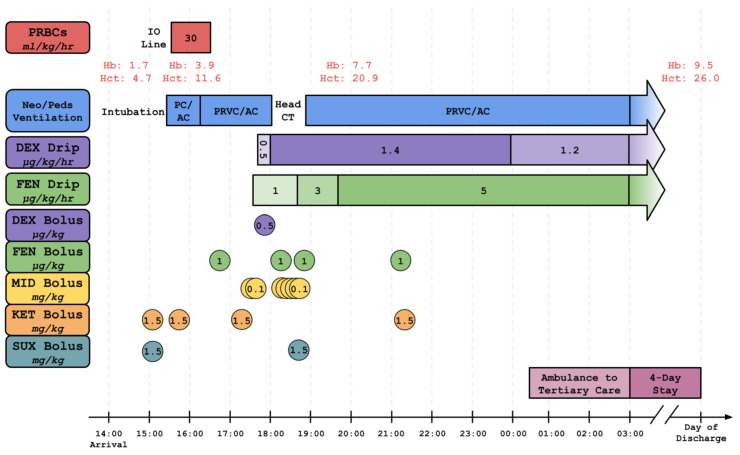
This figure shows a timeline of the patient’s treatment with mechanical ventilation, massive transfusion, sedatives, paralytics, and analgesia, along with important labs, with an emphasis on the stay in the emergency department of the community hospital. Abbreviations: assist control (AC), dexmedetomidine (DEX), fentanyl (FEN), hemoglobin (Hb), hematocrit (Hct), intraosseous (IO), ketamine (KET), midazolam (MID), Neonatal/Pediatric (Neo/Peds), pressure control (PC), packed red blood cell (PRBC), pressure-regulated volume control (PRVC), succinylcholine (SUX).

The ventilator mode was switched to pressure-regulated volume control (PRVC) for the comfort of the patient and to avoid pulmonary edema associated with reduced lung compliance due to aggressive MT. Repeat confirmatory labs taken during MT showed an improved hemoglobin of 3.9 g/dL and hematocrit of 11.6%. At the end of transfusion, the child became more alert, which was problematic as the child was intubated. Sedation progressively became more difficult as cerebral perfusion improved, requiring repetitive doses of fentanyl, midazolam, ketamine, and succinylcholine. Because of an acceleration of agitation while intubated, a fentanyl drip was started at an initial dose of 1 µg/kg/h. The fentanyl drip was increased gradually to a maximum of 5 µg/kg/h, and in addition, a dexmedetomidine bolus of 0.5 µg/kg followed by a drip at a maximum of 1.4 µg/kg/h was required to ensure sedation following the transfusion. The drips were administered with a syringe-based smart infusion system. The emergency physician requested end-tidal CO_2_, which was attached to the sideport of the endotracheal tube extensor. Respiratory therapy was therefore able to titrate ventilator settings based not only on the patient’s response but also on the end-tidal CO_2_. A timeline of the sedative and paralytic medications with blood transfusion and respiratory management is presented in Figure 1.

The patient remained stable in the ED except for a transient period of agitation brought about by the need to manually ventilate the patient for a computerized tomography (CT) scan of the head, which required additional doses of midazolam, fentanyl, and succinylcholine. Once the CT scan was reported as normal and the child returned to the ED, repeat laboratory data were performed, which revealed correction of the acidosis with a bicarbonate of 15.5 mmol/L and the development of hypokalemia with a potassium of 2.8 mmol/L, which was a result of the correction of the metabolic acidosis. The emergency physician discussed this with the pharmacy and the neonatal intensivist and decided to give a single dose of 0.8 mEq of potassium chloride over 1.5 h. The repeat CBC at the conclusion of the transfusion of 30 mL/kg showed a WBC count of 19,900 per µL, hemoglobin of 7.7 g/dL, hematocrit of 20.9%, and reticulocyte count at 7.4%. Coagulation studies revealed normal international normalized ratio and activated partial thromboplastin time, with evidence of hemolysis with fibrin split products ≥ 20 µg/mL and fibrinogen of 140 mg/dL.

Attempts were made to transfer the patient to a nearby hospital with pediatric intensive care. However, special hematology was not available, and therefore, the patient required transfer to a tertiary care center, which was 2.5 h away. Because of inclement weather, helicopter transport was not possible nor was pediatric emergency transport available from the hospital. Therefore, there was a six-hour period of critical care by the emergency physician with the collaboration of the respiratory therapists and neonatal intensivist, who were in frequent communication. After stabilization, there was an additional four-hour period of management of the patient prior to transfer. During this four-hour period, the emergency physician stayed by the bedside and continued to consult with the respiratory therapists and neonatal intensivist. Transfer to the tertiary care center required maintenance by paramedics, with continuous ventilation and fentanyl and dexmedetomidine drips. The child arrived at the tertiary care center around 13 h after initial presentation to the ED.

The patient was admitted to the pediatric intensive care unit and was kept on the fentanyl and dexmedetomidine drips until the morning, when the fentanyl drip was discontinued before extubation and the dexmedetomidine drip was tapered. The patient was then transferred to the pediatric unit 48 h after arrival at the tertiary care center. Salient laboratory data supported the diagnosis of hemolysis related to secondary AIHA presumed to be induced by infection, with an elevated lactate dehydrogenase of 822 units/L (normal: 140–271 units/L) and a 4+ positive indirect and 4+ positive direct Coombs test. Immunoglobulin (Ig) tests revealed an elevated IgM of 2236 mg/dL (normal: 50–170 mg/dL) and slightly elevated IgA and IgG. The etiology for this sepsis was presumed to be the heavy aspirate growth of *Streptococcus pneumoniae* and moderate aspirate growth of *Haemophilus influenzae*, which were sensitive to ceftriaxone and cefepime. In addition, the patient had a negative aspirate methicillin-resistant *Staphylococcus aureus* antibody test. However, the patient had light aspirate growth of *Enterobacter cloacae* and *Kluyvera ascorbata*. These cultures were from deep respiratory cultures from the endotracheal tube on admission to the community hospital. Additionally, nasal swabs detected rhinovirus/enterovirus and adenovirus infections.

The patient was treated with cefepime and vancomycin for 36 h until blood cultures were negative before being transitioned to amoxicillin/clavulanate, which was prescribed at discharge 4 days after admission to the tertiary care center. Treatment with steroids or immunotherapy, including the use of intravenous immunoglobulin (IVIG), was foregone due to the patient’s rapid improvement and positive response to antibiotics. Subsequent follow-up revealed the absence of any immunologic abnormalities, as well as negative indirect and direct Coombs tests. Whole-genome sequencing was performed and found no positive mutations, ruling out the possibility of inherited causes of hemolysis, such as G6PD deficiency or hemoglobinopathy. Five months after discharge, the patient’s hemoglobin and hematocrit remained stable at 12.1 g/dL and 36.9%, respectively.

## 3. Discussion

Anemia is a common pediatric health problem encountered worldwide, yet presentation with a hemoglobin as low as 1.7 g/dL is rarely reported. Table 2 describes similar cases for pediatric patients with a hemoglobin < 2 g/dL. This case is a rare entity, as there are only 5 other cases of pediatric anemia with a hemoglobin < 2 g/dL reported in the literature. Moreover, there is only one other case of pediatric AIHA with this low a hemoglobin reported [5].

This patient was presumed to have secondary AIHA triggered by infection, as evidenced by the positive bacterial cultures taken from the endotracheal tube and rhinovirus/enterovirus and adenovirus infections taken from nasal swabs. AIHA is characterized by the body’s production of autoantibodies that target red blood cells and cause their destruction [13]. Due to the patient’s hypothermia and greatly elevated IgM, it is theorized that she had the cold subtype of AIHA, also known as secondary cold agglutinin syndrome, which is characterized by cold agglutinins binding to red blood cell antigens at temperatures lower than body temperature [13,14]. It has been observed in the literature that cold AIHA most often does not require treatment with steroids or IVIG, which may explain why the patient improved after treatment with massive transfusion and antibiotics [14]. However, since neither a monospecific direct antiglobulin test for complement C3d nor a cold agglutinin titer were run in addition to the polyspecific Coombs test, the exact pathophysiology of the AIHA cannot be determined with certainty [13]. Therefore, the patient’s presumed diagnosis of secondary AIHA due to bacterial and viral infection remains probable rather than definitive. However, the absence of hemoglobinuria makes paroxysmal cold hemoglobinuria less likely, even though the Donath–Landsteiner test was not done. Subsequent genetic testing was negative for mutations causing hemoglobinopathies or other hereditary blood disorders. Therefore, the return to normal development and hematologic physiology at five months after discharge further supports the diagnosis of secondary AIHA.

The success of treating this patient in the community hospital depended on interprofessional collaboration to overcome inefficiencies inherent to communication within and between hospital departments. There were four main areas where collaboration was necessary for the ED to successfully manage this rare and life-threatening child in sepsis with severe anemia: 1. Management of the airway and ventilator to avoid barotrauma, which was coordinated by the respiratory therapists and emergency physician; 2. Placement of an IO line with a 45-mm needle, which is usually reserved for adults; 3. Administration of MT and correction of potassium deficiency; and 4. Repetitive doses of analgesia and sedation with fentanyl and dexmedetomidine drips.

PRBCs have a high viscosity and a low flow rate, so the use of a 15-gauge IO needle allowed for more rapid transfusion of PRBCs than the 22-gauge IV catheter placed on admission [15]. The emergency physician elected to use the adult, 45-mm IO needle in the distal femur proximal to the femoral diaphysis because of the higher likelihood of establishment of functional high-flow IO access due to greater stability from deeper medullary bone placement, with a greater success rate than the traditional pediatric, 15-mm needles [16,17]. The use of an adult IO needle for an 8-kg pediatric patient’s resuscitation is considered “off-label” since the patient was not the intended age, so proper placement was necessary to avoid going completely through the femur, particularly in a small child with low body mass.

Outside of the trauma and surgical settings, MT in a pediatric patient is a rare event, which can be quite challenging for an ED in a community hospital, particularly when associated with respiratory failure, severe sepsis, and life-threatening anemia [18]. There have been multiple definitions of pediatric MT in the literature, leading to differing opinions about what can be considered MT. While some have defined pediatric MT as replacement of 50% of the child’s total blood volume, others have used 20 or 40 mL/kg as lower thresholds for defining MT [18,19,20,21]. It has been recently proposed that any pediatric transfusion greater than 20 mL/kg within one hour should be considered MT, since these children have a high mortality risk and an urgent need for intervention [21]. Therefore, this patient, who received 30 mL/kg PRBCs in an hour, would meet the threshold for MTP activation under certain definitions.

It is standard to transfuse anemic pediatric patients at a maximum of 15 mL/kg over 3 to 4 h, since excessive transfusion of PRBCs in a pediatric patient can lead to TACO [6,22]. However, it is also recommended that septic children are transfused to a hemoglobin of at least 7 g/dL [22,23]. It is estimated that the transfusion of 5 mL/kg PRBCs increases hemoglobin by 1 g/dL in children, which meant that this patient with a hemoglobin of 1.7 g/dL would require much more than 15 mL/kg to become stabilized [24,25]. In this setting, the emergency physician was faced with a decision whether to fully transfuse this patient up to a hemoglobin of 7 g/dL or transfuse less to avoid the possibility of TACO. Therefore, the emergency physician initially transfused 120 mL (15 mL/kg), at which point consultation with the neonatal intensivist allowed for the completion of the remaining 120 mL, leading to a final transfusion volume of 240 mL PRBCs. This child did not develop TACO nor suffer renal insufficiency because of prolonged shock. There was a risk in administration of such a high volume and rate, yet the transfusion was successful, as evidenced by the attainment of a hemoglobin of 7.7 g/dL, the return of vigorous motor activity of the patient, and the need for increased sedation at the end of the transfusion. While the literature cautions for judicious use of MT for severely anemic patients, judicious administration of 30 mL/kg blood transfusion has been demonstrated to be successful in such severely ill children [26]. The decision regarding the volume and speed at which MT is provided for this severely anemic pediatric patient required individual assessment by the emergency physician and neonatal intensivist, given the possible complications of TACO.

Correction of the acidosis through increased tissue perfusion with oxygenated hemoglobin uncovered a total potassium deficit with a post-transfusion potassium of 2.8 mmol/L. The initial acidosis caused an efflux of potassium from the intracellular space to the extracellular space, and the correction of the acidosis with transfusion and the improvement of serum bicarbonate led to a shift of potassium back into the cell, which is a common occurrence in acidosis [27,28,29]. Correction of hypokalemia was deemed necessary prior to the lengthy transport, and the emergency physician relied on the neonatal intensivist to determine a safe concentration and rate of the potassium bolus.

In addition to providing guidance on the concentration and frequency of the boluses of sedatives and analgesia, the neonatal intensivist also assisted the emergency physician in determining what drips were necessary and at what rate to give them. However, this proved to be a challenge, as several ED nurses were unfamiliar with the syringe-based smart infusion system, which is commonly used for pediatric and neonatal patients [30]. Thus, senior ED nurses and neonatal intensive care nurses stayed three hours after their shifts had ended to guide other nurses in the use of the infusion system and the management of the drips for successful sedation.

**Figure 2:**
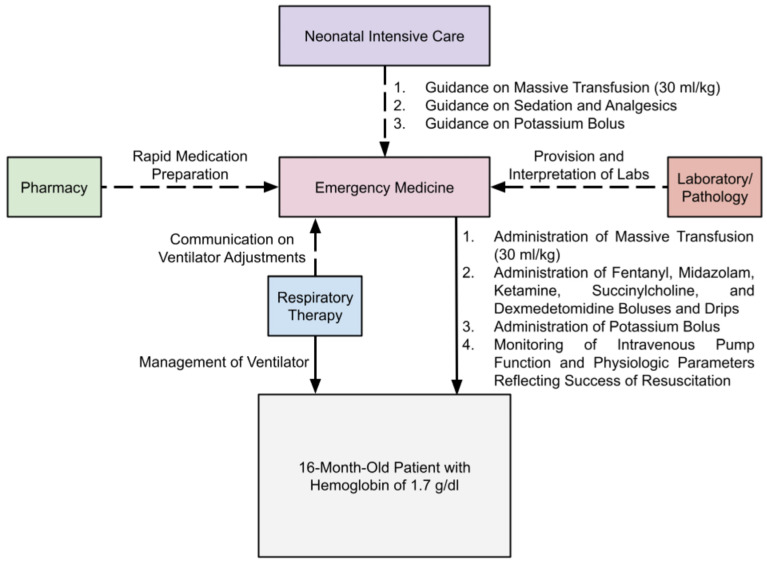
This figure shows the collaboration between different departments with the emergency department to provide care for the patient. The dashed arrows show that the communication between departments was frequent and indirectly beneficial to the patient, while the solid arrows represent the constant, direct care provided to the patient because of effective collaboration.

Throughout the resuscitation in the community hospital, the emergency physician remained continuously at the bedside coordinating care, troubleshooting equipment challenges, and integrating input from nursing, pharmacy, respiratory therapy, laboratory, pathology, and neonatal critical care (Figure 2). Frequent consultation with the neonatal intensivist allowed for safe resuscitation of the child without the presence of a pediatric intensivist. The continuity of care and a smooth handoff between changing shifts—entailing more collaboration within and outside the department—were critical for ensuring stable, continuous care that allowed for the patient’s survival. This case illustrates that survival in extreme pediatric anemia in resource-limited settings may hinge as much on human factors, equipment familiarity, and professional collaboration as on medical decision-making alone.

## 4. Conclusions

This case highlights the challenges of managing profound anemia and severe sepsis in a young child within the ED of a community hospital without the assistance of a pediatric intensivist. Survival depended not just on a single intervention but on sustained collaboration between and within departments, adaptive use of available resources, and continuity of critical care.

## Figures and Tables

**Table 1:** This table displays the laboratory values at three different times on the day the patient was initially admitted to the emergency department of the community hospital and once two days prior to discharge from the tertiary care center.

Laboratory Value (Reference Range)	Day 1 14:37	Day 1 15:51	Day 1 20:00	Day 4 4:28
Complete Blood Count				
White Blood Cells (5.0–17.0 thousand/µL)	59.7	56.5	19.9	7.4
Red Blood Cells (3.4–5.2 million/µL)	0.32	0.89	1.74	2.28
Hematocrit (35.0–42.0%)	4.7	11.6	20.9	26.0
Hemoglobin (9.9–14.5 g/dL)	1.7	3.9	7.7	9.5
Mean Corpuscular Volume (70–96 fL)	147.3	130.6	119.8	114
Mean Platelet Volume (7.2–11.5 fL)	7.3	6.7	6.4	7.9
Platelets (130–400 thousand/µL)	463	341	258	155
Chemical Profile				
Anion Gap (6–22)	36	35	16	8
Total Bilirubin (0–1 mg/dL)	1.4	0.8	0.7	1.5
Bilirubin (Indirect) (0.0–0.3 mg/dL)	0.7			
CO_2_ (21–29 mmol/L)	6	4	15	29
C-Reactive Protein (0.0–3.0 mg/L)	30.4			
Glucose (70–105 mg/dL)	22	112	146	71
Potassium (3.6–5.2 mmol/L)	4.8	5	2.8	4.4
Arterial Blood Gas				
pH, Arterial (7.37–7.44)	7.09		7.35	
O_2_ Partial Pressure, Arterial (80–110 mmHg)	144		108	
CO_2_ Partial Pressure, Arterial (31–45 mmHg)	13		28	
Bicarbonate, Arterial (22–26 mmol/L)	6		15.5	
Coagulation Studies				
Activated Partial Thromboplastin Time (23–34 s)			26.5	
Fibrinogen (180–350 mg/dL)			140	
International Normalized Ratio (≤4.9)			1.2	
Sepsis Markers				
Procalcitonin (0–0.09 ng/mL)	0.71			
Reticulocyte Count (0.6–2.4%)		9.3	7.4	
Fibrin Split Products (<5 µg/mL)			≥20	
Prothrombin Time (9.0–12.0 s)			13.5	

**Table 2:** This table displays information about reports in the literature of pediatric patients with a hemoglobin < 2 g/dL. Abbreviations: autoimmune hemolytic anemia (AIHA), iron deficiency anemia (IDA), packed red blood cell (PRBC).

Case	Year	Country	Age (Months)	Initial Hemoglobin (g/dL)	Transfusion Amount	Diagnosis	Resultant Hemoglobin (g/dL)
Present Case	2026	United States	16	1.7	30 mL/kg PRBCs	AIHA	12.1 (at 5 months)
Gutata [11]	2024	Ethiopia	18	0.8	1 unit whole blood	Malaria	12.0 (at 5 days)
Shalby et al. [8]	2022	Saudi Arabia	20	1.1	10 mL/kg PRBCs	Nutritional IDA	6.0–8.0 (at 10 months)
Shalby et al. [8]	2022	Saudi Arabia	6	1.2	“Multiple” Blood Transfusions	Nutritional IDA	7.4 (at Discharge at Unknown Time)
Hanna and Carcao [5]	2023	Canada	~72	1.6	≥1 unit PRBCs	Mixed AIHA	10.7 (at 110 days)
Parodi et al. [12]	2021	Italy	~60	1.9	15 mL/kg PRBCs	Mixed IDA and Homozygous Sickle Cell Disease	9.8 (at 10 days)

## Data Availability

The data presented in this study are available on reasonable request from the corresponding author due to privacy and ethical concerns.

## Abbreviations

The following abbreviations are used in this manuscript:

AC	Assist control
AIHA	Autoimmune hemolytic anemia
CBC	Complete blood count
CT	Computerized tomography
ED	Emergency department
DEX	Dexmedetomidine
FEN	Fentanyl
Hb	Hemoglobin
Hct	Hematocrit
IDA	Iron deficiency anemia
Ig	Immunoglobulin
IO	Intraosseous
IV	Intravenous
IVIG	Intravenous immunoglobulin
KET	Ketamine
MCV	Mean corpuscular volume
MID	Midazolam
MT	Massive transfusion
MTP	Massive transfusion protocol
Neo/Peds	Neonatal/Pediatric
PC	Pressure control
PRBC	Packed red blood cell
PRVC	Pressure-regulated volume control
SUX	Succinylcholine
TACO	Transfusion-associated circulatory overload
